# Somatic instability of the *FGF14*-SCA27B GAA•TTC repeat reveals a marked expansion bias in the cerebellum

**DOI:** 10.1093/brain/awae312

**Published:** 2024-10-08

**Authors:** David Pellerin, Jean-Loup Méreaux, Susana Boluda, Matt C Danzi, Marie-Josée Dicaire, Claire-Sophie Davoine, David Genis, Guinevere Spurdens, Catherine Ashton, Jillian M Hammond, Brandon J Gerhart, Viorica Chelban, Phuong U Le, Maryam Safisamghabadi, Christopher Yanick, Hamin Lee, Sathiji K Nageshwaran, Gabriel Matos-Rodrigues, Zane Jaunmuktane, Kevin Petrecca, Schahram Akbarian, André Nussenzweig, Karen Usdin, Mathilde Renaud, Céline Bonnet, Gianina Ravenscroft, Mario A Saporta, Jill S Napierala, Henry Houlden, Ira W Deveson, Marek Napierala, Alexis Brice, Laura Molina Porcel, Danielle Seilhean, Stephan Zuchner, Alexandra Durr, Bernard Brais

**Affiliations:** Dr John T. Macdonald Foundation Department of Human Genetics and John P. Hussman Institute for Human Genomics, University of Miami Miller School of Medicine, Miami, FL 33136, USA; Department of Neurology and Neurosurgery, Montreal Neurological Hospital and Institute, McGill University, Montreal, QC H3A 2B4, Canada; Department of Neuromuscular Diseases, UCL Queen Square Institute of Neurology and The National Hospital for Neurology and Neurosurgery, University College London, London WC1N 3BG, UK; Sorbonne Université, Institut du Cerveau—Paris Brain Institute- ICM, Inserm, CNRS, APHP, University Hospital Pitié-Salpêtrière, F-75013 Paris, France; Sorbonne Université, Institut du Cerveau—Paris Brain Institute- ICM, Inserm, CNRS, APHP, University Hospital Pitié-Salpêtrière, F-75013 Paris, France; Dr John T. Macdonald Foundation Department of Human Genetics and John P. Hussman Institute for Human Genomics, University of Miami Miller School of Medicine, Miami, FL 33136, USA; Department of Neurology and Neurosurgery, Montreal Neurological Hospital and Institute, McGill University, Montreal, QC H3A 2B4, Canada; Sorbonne Université, Institut du Cerveau—Paris Brain Institute- ICM, Inserm, CNRS, APHP, University Hospital Pitié-Salpêtrière, F-75013 Paris, France; Ataxia and Hereditary Spastic Paraplegia Unit, Service of Neurology, Hospital Universitari de Girona Dr. Josep Trueta (ICS) & Hospital Santa Caterina IAS, Institut d’Investigació Biomèdica de Girona (IDIBGI), 17007 Girona, Spain; Dr John T. Macdonald Foundation Department of Human Genetics and John P. Hussman Institute for Human Genomics, University of Miami Miller School of Medicine, Miami, FL 33136, USA; Department of Neurology and Neurosurgery, Montreal Neurological Hospital and Institute, McGill University, Montreal, QC H3A 2B4, Canada; Department of Neurology, Royal Perth Hospital, Perth, WA 6000, Australia; Genomics and Inherited Disease Program, Garvan Institute of Medical Research, Sydney, NSW 2010, Australia; Centre for Population Genomics, Garvan Institute of Medical Research and Murdoch Children’s Research Institute, Sydney, NSW 2010, Australia; Department of Neurology, Peter O’Donnell Jr. Brain Institute, University of Texas Southwestern Medical Center, Dallas, TX 75390-8823, USA; Department of Neuromuscular Diseases, UCL Queen Square Institute of Neurology and The National Hospital for Neurology and Neurosurgery, University College London, London WC1N 3BG, UK; Neurobiology and Medical Genetics Laboratory, ‘Nicolae Testemitanu’ State University of Medicine and Pharmacy, MD-2004 Chisinau, Republic of Moldova; Department of Neurology and Neurosurgery, Montreal Neurological Hospital and Institute, McGill University, Montreal, QC H3A 2B4, Canada; Department of Neurology and Neurosurgery, Montreal Neurological Hospital and Institute, McGill University, Montreal, QC H3A 2B4, Canada; Department of Neurology, University of Miami Miller School of Medicine, Miami, FL 33136, USA; Department of Neuromuscular Diseases, UCL Queen Square Institute of Neurology and The National Hospital for Neurology and Neurosurgery, University College London, London WC1N 3BG, UK; Department of Psychiatry, Friedman Brain Institute Icahn School of Medicine at Mount Sinai, New York, NY 10029-5674, USA; Department of Neuroscience, Friedman Brain Institute Icahn School of Medicine at Mount Sinai, New York, NY 10029-5674, USA; Department of Genetics and Genomic Sciences, Friedman Brain Institute Icahn School of Medicine at Mount Sinai, New York, NY 10029-5674, USA; Neurogenetics Program, Department of Neurology, David Geffen School of Medicine, University of California Los Angeles, Los Angeles, CA 90095, USA; Laboratory of Genome Integrity, National Cancer Institute, NIH, Bethesda, MD 20892, USA; Division of Neuropathology, The National Hospital for Neurology and Neurosurgery, University College London NHS Foundation Trust, London WC1N 3BG, UK; Department of Clinical and Movement Neurosciences, UCL Queen Square Institute of Neurology, University College London, London WC1N 3BG, UK; Queen Square Brain Bank for Neurological Disorders, UCL Queen Square Institute of Neurology, University College London, London WC1N 3BG, UK; Department of Neurology and Neurosurgery, Montreal Neurological Hospital and Institute, McGill University, Montreal, QC H3A 2B4, Canada; Department of Psychiatry, Friedman Brain Institute Icahn School of Medicine at Mount Sinai, New York, NY 10029-5674, USA; Department of Neuroscience, Friedman Brain Institute Icahn School of Medicine at Mount Sinai, New York, NY 10029-5674, USA; Department of Genetics and Genomic Sciences, Friedman Brain Institute Icahn School of Medicine at Mount Sinai, New York, NY 10029-5674, USA; Laboratory of Genome Integrity, National Cancer Institute, NIH, Bethesda, MD 20892, USA; Laboratory of Cell and Molecular Biology, National Institute of Diabetes and Digestive and Kidney Diseases, National Institutes of Health, Bethesda, MD 20892, USA; INSERM-U1256 NGERE, Université de Lorraine, 54500 Nancy, France; Service de Neurologie, CHRU de Nancy, 54000 Nancy, France; Service de Génétique Clinique, CHRU de Nancy, 54000 Nancy, France; INSERM-U1256 NGERE, Université de Lorraine, 54500 Nancy, France; Laboratoire de Génétique, CHRU de Nancy, 54000 Nancy, France; Harry Perkins Institute of Medical Research, Centre for Medical Research, University of Western Australia, Nedlands, Perth, WA 6009, Australia; Department of Neurology, University of Miami Miller School of Medicine, Miami, FL 33136, USA; Department of Neurology, Peter O’Donnell Jr. Brain Institute, University of Texas Southwestern Medical Center, Dallas, TX 75390-8823, USA; Department of Neuromuscular Diseases, UCL Queen Square Institute of Neurology and The National Hospital for Neurology and Neurosurgery, University College London, London WC1N 3BG, UK; Genomics and Inherited Disease Program, Garvan Institute of Medical Research, Sydney, NSW 2010, Australia; Centre for Population Genomics, Garvan Institute of Medical Research and Murdoch Children’s Research Institute, Sydney, NSW 2010, Australia; Faculty of Medicine, University of New South Wales, Sydney, NSW 2052, Australia; Department of Neurology, Peter O’Donnell Jr. Brain Institute, University of Texas Southwestern Medical Center, Dallas, TX 75390-8823, USA; Sorbonne Université, Institut du Cerveau—Paris Brain Institute- ICM, Inserm, CNRS, APHP, University Hospital Pitié-Salpêtrière, F-75013 Paris, France; Alzheimer’s Disease and other Cognitive Disorders Unit, Service of Neurology, Hospital Clínic, Fundació de Recerca Clínic Barcelona-Institut d’Investigacions Biomediques August Pi i Sunyer (FRCB-IDIBAPS), University of Barcelona, 08036 Barcelona, Spain; Neurological Tissue Brain Bank, Biobanc-Hospital Clínic-FRCB-IDIBAPS, 08036 Barcelona, Spain; Sorbonne Université, Institut du Cerveau—Paris Brain Institute- ICM, Inserm, CNRS, APHP, University Hospital Pitié-Salpêtrière, F-75013 Paris, France; Dr John T. Macdonald Foundation Department of Human Genetics and John P. Hussman Institute for Human Genomics, University of Miami Miller School of Medicine, Miami, FL 33136, USA; Sorbonne Université, Institut du Cerveau—Paris Brain Institute- ICM, Inserm, CNRS, APHP, University Hospital Pitié-Salpêtrière, F-75013 Paris, France; Department of Neurology and Neurosurgery, Montreal Neurological Hospital and Institute, McGill University, Montreal, QC H3A 2B4, Canada; Department of Human Genetics, McGill University, Montreal, QC H3A 0C7, Canada

**Keywords:** SCA27B, spinocerebellar ataxia 27B, GAA-FGF14 ataxia, repeat expansion disorder, mosaicism, repeat expansion

## Abstract

Spinocerebellar ataxia 27B (SCA27B) is a common autosomal dominant ataxia caused by an intronic GAA•TTC repeat expansion in *FGF14*. Neuropathological studies have shown that neuronal loss is largely restricted to the cerebellum. Although the repeat locus is highly unstable during intergenerational transmission, it remains unknown whether it exhibits cerebral mosaicism and progressive instability throughout life.

We conducted an analysis of the *FGF14* GAA•TTC repeat somatic instability across 156 serial blood samples from 69 individuals, fibroblasts, induced pluripotent stem cells and post-mortem brain tissues from six controls and six patients with SCA27B, alongside methylation profiling using targeted long-read sequencing. Peripheral tissues exhibited minimal somatic instability, which did not significantly change over periods of more than 20 years. In post-mortem brains, the GAA•TTC repeat was remarkably stable across all regions, except in the cerebellar hemispheres and vermis. The levels of somatic expansion in the cerebellar hemispheres and vermis were, on average, 3.15 and 2.72 times greater relative to other examined brain regions, respectively. Additionally, levels of somatic expansion in the brain increased with repeat length and tissue expression of *FGF14*. We found no significant difference in methylation of wild-type and expanded *FGF14* alleles in post-mortem cerebellar hemispheres between patients and controls.

In conclusion, our study revealed that the *FGF14* GAA•TTC repeat exhibits a cerebellar-specific expansion bias, which may explain the pure cerebellar involvement in SCA27B.

## Teaser

Spinocerebellar ataxia 27B (SCA27B) is a common ataxia caused by a GAA•TTC repeat expansion in *FGF14*. Pellerin, Méreaux *et al.* show that this repeat is stable in most tissues and brain regions but expands specifically in the cerebellum, possibly explaining the pure cerebellar involvement in SCA27B.

## Introduction

Spinocerebellar ataxia 27B (SCA27B; GAA-*FGF14*-related ataxia) is a recently described autosomal dominant ataxia caused by the expansion of a polymorphic GAA•TTC repeat located within intron 1 of the fibroblast growth factor 14 (*FGF14*) gene.^1,2^ SCA27B is phenotypically characterized by a late-onset, slowly progressive pan-cerebellar syndrome that is frequently associated with episodic symptoms and downbeat nystagmus.^1,3-5^ The size of the GAA•TTC repeat expansion only shows a weak negative correlation with the age at onset^1,6^ and has not been associated with disease severity or progression.^3,4^ Furthermore, there exists significant phenotypic variability among patients carrying expansions of similar sizes, suggesting that additional factors influence disease expressivity.^3,4,6^ Neuropathological studies in SCA27B have shown that neuronal loss is largely restricted to the cerebellar cortex, involving the vermis more than the hemispheres.^1,3^ However, it is noteworthy that significant pathological abnormalities are not found in other regions despite the widespread expression of *FGF14* in the brain, albeit at lower levels than in the cerebellum.^7^ This observation suggests that tissue-specific factors may increase the vulnerability of the cerebellum in SCA27B.

Although the expanded *FGF14* GAA•TTC repeat is highly unstable upon intergenerational transmission,^4,8^ whether it exhibits somatic instability in the brain has not yet been investigated. Somatic mosaicism is a phenomenon observed in many repeat expansions, which have the ability to form alternative (non-B) DNA structures that predispose them to errors during DNA repair.^9-11^ The degree of somatic instability generally increases with the length of the repeat tract and age, and also shows a repeat-specific pattern of variation across different tissues and cell types.^12^ Remarkably, previous studies on coding CAG•CTG repeat expansions in SCA1, SCA2, SCA3, SCA7, and dentatorubral pallidoluysian atrophy (DRPLA) have shown that these diseases consistently have smaller expansion sizes and lower degrees of mosaicism in the cerebellum compared to other brain regions.^13-17^ In comparison, the intronic ATTTC pentanucleotide repeat insertion in *DAB1* exhibits longer sizes and an expansion bias in the cerebellum compared to blood samples and fibroblasts in patients with SCA37.^18^ The intronic *RFC1* AAGGG•CCCTT repeat expansion, however, shows little instability in the cerebellum of patients with cerebellar ataxia, neuropathy, and vestibular areflexia syndrome (CANVAS).^19^ Furthermore, in Friedreich's ataxia, although the expanded intronic *FXN* GAA•TTC repeat is longer in the cerebellum than in other brain regions, large contractions occur significantly more frequently than large expansions.^20-23^

Here, to gain further insight into SCA27B biology, we performed a comprehensive analysis of the *FGF14* GAA•TTC repeat somatic instability in serial blood samples, fibroblasts, induced pluripotent stem cells (iPSCs) and post-mortem brains from individuals with SCA27B.

## Materials and methods

Detailed methods are provided in the Supplementary material.

### Human sample collection

Human post-mortem brain tissue was collected from six non-ataxic control individuals and six persons with SCA27B to study the somatic instability of the *FGF14* GAA•TTC repeat tract (Table 1). Fresh frozen samples were obtained from various regions of the CNS, as detailed in Supplementary Table 1. Additionally, DNA extracted from blood and cerebellar hemispheres of another eight control individuals was obtained to compare allele sizes between both tissues. We also collected fresh frozen tissue of the cerebellar hemispheres from an additional four controls and one SCA27B patient to perform methylation profiling of *FGF14* with Oxford Nanopore Technologies (ONT), as detailed in the Supplementary material, ‘Methods’ section.

**Table 1:** Demographic and genetic characteristics of the post-mortem brain samples analysed for *FGF14* GAA•TTC repeat instability

Study site	Status	ID number	Sex	GAA•TTC repeat units	Age at onset (years)	Age at death (years)
Montreal	Control	C1	M	15/92	–	76
Montreal	Control	C2	M	9/36	–	68
Paris	Control	C3	M	16/152	–	70
Paris	Control	C4	F	23/165	–	92
Paris	Control	C5	M	47/151	–	82
Paris	Control	C6	M	17/42	–	66
Montreal	SCA27B	P1	M	292/304	52	69
Barcelona	SCA27B	P2	M	9/288	71	91
Barcelona	SCA27B	P3	F	191/248	78	86
Paris	SCA27B	P4	M	31/548	46	72
Paris	SCA27B	P5	F	70/480	NA	85
Paris	SCA27B	P6	M	32/405	50	78

F = female; M = male; NA = not available; SCA27B = spinocerebellar ataxia 27B.

### Analysis of somatic instability

**Figure 1:**
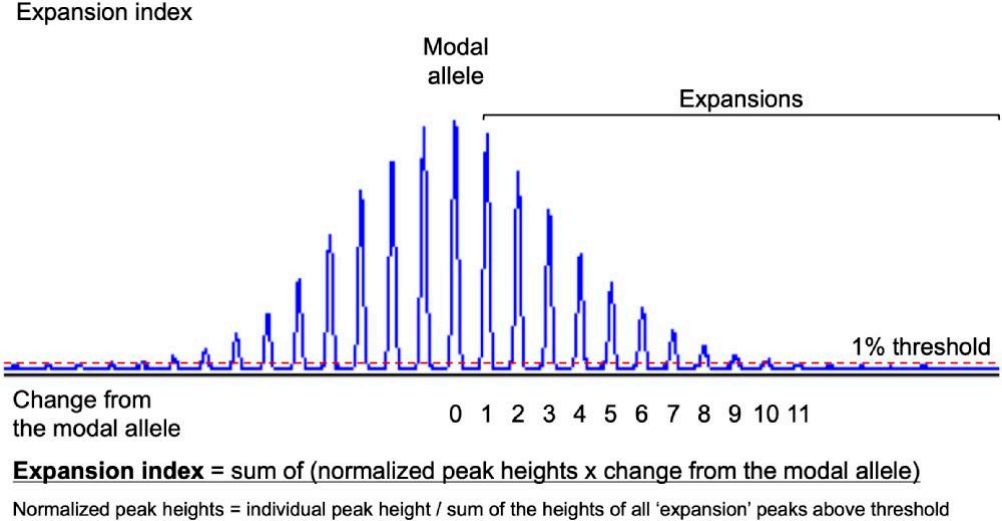
**Determination of the *FGF14* GAA•TTC repeat expansion index**. Method for calculating the expansion index of the *FGF14* GAA•TTC repeat. The expansion index is calculated by only taking into account peaks to the right of the modal allele, which represent somatically expanded GAA•TTC repeats, and using a 1% relative peak threshold. The modal allele corresponds to the peak with the highest intensity, as measured in relative fluorescence units in GeneMapper or Peak Scanner. The expansion index is calculated by first dividing the height of each individual peak by the sum of the heights of all peaks to the right of the modal allele that are above the set 1% threshold. The resulting normalized peak heights are then multiplied by the position of the peak (change from the modal allele) and these values are summed to generate the index.

Somatic instability was studied by capillary electrophoresis or by agarose gel electrophoresis for samples carrying alleles >400–450 triplets. For samples analysed by capillary electrophoresis, the GeneMapper software v6.0 (Applied Biosystems) or Peak Scanner software v1.0 (Applied Biosystems) was used to establish the ‘modal peak’ of each of the two alleles in each sample, which was defined as the peak with the highest height. Peaks to the right of the modal allele represent somatically expanded GAA•TTC repeats, while peaks to the left may include both contracted GAA•TTC repeats and PCR stutter products. Expansion indices (EIs) were calculated by only taking into account peaks to the right of the ‘modal allele’ and using a 1% relative peak threshold (Fig. 1), as described previously.^13,24^ The EI was obtained by first dividing the height of each individual peak by the sum of the heights of all peaks above the threshold. The resulting normalized peak heights were then multiplied by the position of the peak and these normalized peak values were summed to generate the EI. Analysis of blood and CNS tissues revealed that the modal allele length was remarkably stable across all regions examined, except in the cerebellar hemispheres and vermis where it showed a tendency to expand. We therefore considered the modal allele length as measured in the blood and most CNS regions as the constitutional allele length, which we set as the main allele for calculation of the EI in tissues with an expansion shift of the modal repeat,^13^ tolerating a discrepancy of one triplet due to the variability of our genotyping assay.^25^ Finally, we correlated the somatic instability indices with the expression of *FGF14* in normal tissue data from the Human Protein Atlas (HPA v.23.0; proteinatlas.org accessed 27 March 2024).^26^

### Ethics

The institutional review boards of the Montreal Neurological Hospital, Montreal (MPE-CUSM-15-915), the Centre Hospitalier de l’Université de Montréal, Montreal (ND02.045), the Dr Josep Trueta University Hospital, Girona (2022.120), the Hôpital Pitié-Salpêtrière, Paris (SPATAX RBM 01-29 and RBM 03-48, BIOMOV NCT05034172), and the University College London Hospitals, London (04/N034) approved this study. All brain donors signed an informed consent for brain tissue usage in medical research. The study complied with all relevant ethical regulations.

## Results

### Longitudinal analysis of the *FGF14* GAA•TTC repeat somatic instability in blood samples

**Figure 2:**
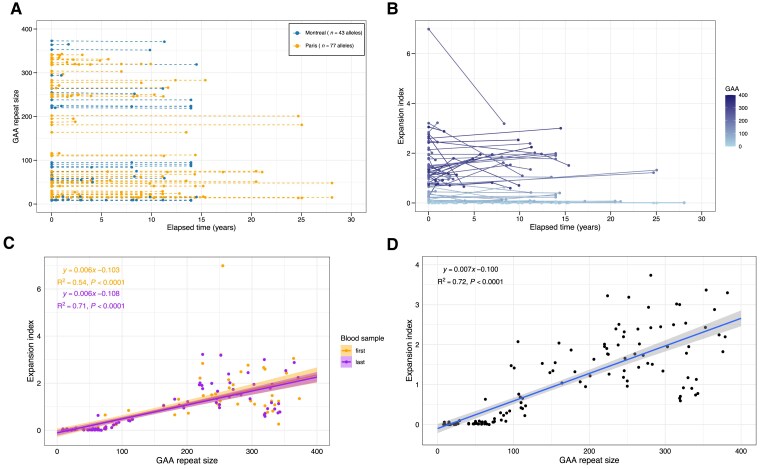
**Longitudinal analysis of the *FGF14* GAA•TTC repeat somatic instability in blood samples**. Longitudinal analysis of (**A**) the *FGF14* GAA•TTC repeat tract size, expressed in triplet repeat counts, and (**B**) expansion index across 69 individuals (120 alleles analysed) who underwent serial blood collections over a median period of 8.9 years (interquartile range: 2.9–13.9). Observations from the same person are connected by a line. In **B**, the colour gradient shows the GAA•TTC allele size of each data point. (**C**) Positive linear relationship between the *FGF14* GAA•TTC repeat tract size, expressed in triplet repeat counts, and expansion index in each of the first and last longitudinal blood samples across 69 individuals (*n* = 120 alleles). The Pearson’s correlation coefficient is r = 0.73 [95% confidence interval (CI): 0.64–0.81] for the first blood samples and r = 0.84 (95% CI: 0.78–0.89) for the last blood samples. Both regression lines did not differ significantly (mixed-effect analysis, *P* = 0.72), indicating relative stability of the expansion index over time. (**D**) Positive linear relationship between the *FGF14* GAA•TTC repeat tract size and expansion index across 100 individuals (*n* = 173 alleles), including the results from the last blood samples of the 69 individuals featured in **B** and **C**. The Pearson’s correlation coefficient is r = 0.85 (95% CI: 0.80–0.88). In **C** and **D**, the shaded areas display the 95% CI.

We performed a longitudinal analysis to determine whether the length of the *FGF14* repeat tracts changes over time in 156 serial blood samples from 69 individuals (see Supplementary material, ‘Results’ section). Alleles longer than ≈400–450 triplets could not be measured by capillary electrophoresis and were not included in this analysis. The median interval between the first and last blood sample was 8.9 years [interquartile range (IQR): 2.9–13.9]. We observed no significant change of the modal *FGF14* repeat size between the first and last blood samples [Fig. 2A; median difference (size of last sample − size of first sample), 0 triplet; IQR: 0–0; Sign test, *P* = 0.22] and found that elapsed time had no significant effect on the modal repeat size as assessed by a linear mixed-effects model [estimate = −0.441, standard error (SE) = 0.678, *P* = 0.52].

As for repeat sizes, the EI did not significantly change over time in repeated blood samples [Fig. 2B and C; median of differences (EI of last sample − EI of first sample), 0 (IQR: −0.02–0.01); Sign test, *P* = 0.22], nor in the subset of alleles longer than 200 triplets [median of differences, 0 (IQR: −0.28–0.43); Sign test, *P* = 1]. In a linear mixed-effects model, we found that allele size (estimate = 5.530 × 10^−03^, SE = 3.253 ×10^−04^, *P* < 0.0001) had a significant effect on the EI, while elapsed time (estimate = −7.634 ×10^−03^, SE = 6.945 ×10^−03^, *P* = 0.27), the interaction between allele size and elapsed time (estimate = 9.008 × 10^−05^, SE = 4.839 × 10^−05^, *P* = 0.064), and sex (estimate = −5.148 × 10^−02^, SE = 9.654 × 10^−02^, *P* = 0.60) did not. Regression analysis conducted on 173 alleles from 100 individuals (the EI of alleles longer than ≈400–450 triplets could not be adequately determined by capillary electrophoresis and were not included in this analysis) confirmed a linear increase in somatic expansion with *FGF14* GAA•TTC repeat size [r = 0.85 (95% confidence interval, CI: 0.80 to 0.88), slope = 0.007, *P* < 0.0001] (Fig. 2D). Furthermore, we compared the size and EI of the *FGF14* repeats in two pairs of monozygotic twins with SCA27B who had blood samples drawn at the age of 67 and 64 years, respectively. We observed a difference of a single triplet between them (15/225 versus 15/226 and 9/427 versus 9/428 triplets, respectively), which falls within the expected range of variability of our genotyping assay.^25^ The short alleles of both pairs of twins did not exhibit somatic expansion (EI = 0), while the EI of the long allele of the first pair of twins differed by only 0.5 (EI = 1.76 versus 1.26; the EI of the long allele of the second pair of twins could not be calculated due to their large size). Overall, our results demonstrate that *FGF14* GAA•TTC repeat size and somatic expansion in blood samples remain largely stable over periods of more than 20 years.

### Instability of the *FGF14* GAA•TTC repeat tract in fibroblasts and induced pluripotent stem cells

To further assess somatic instability of the *FGF14* GAA•TTC repeat in peripheral tissues, we analysed allele sizes in paired blood and fibroblasts obtained from three patients with SCA27B. These patients respectively carried alleles of 292/304 (patient P1), 9/508 (patient P8) and 16/389 (patient P9) repeat units, as measured in blood samples. We found minimal difference in the number of GAA•TTC repeats and EI between paired blood and fibroblasts, providing further evidence for the lack of significant instability of the *FGF14* repeat locus in peripheral tissues (Supplementary Figs 4 and 5 and Supplementary Table 2). Moreover, the sizes of both wild type and expanded alleles remained unchanged across ten passages in fibroblasts from two SCA27B patients (patient P8: 9/518 GAA•TTC repeats; patient P9: 16/409 GAA•TTC repeats).

Reprogramming of fibroblasts into iPSCs resulted in variable increase of the modal peak for most expanded alleles but not wild-type alleles (Supplementary Figs 4 and 5 and Supplementary Table 2). While two iPSC clones from patient P1 exhibited minimal changes in modal alleles compared to fibroblasts, one clone harboured an expansion of 11 triplets of its longer allele, corresponding to an increase in length of 3.6% (Supplementary Fig. 6). During reprogramming, the longer allele in all four iPSC clones from patient P9 remained relatively stable (modal difference ranging from −19 to +2 triplets; Supplementary Fig. 4), while it expanded by 7 to 89 triplets in iPSC clones from patient P8 (Supplementary Fig. 5). Finally, similar to fibroblasts, the sizes of alleles in control (*n* = 3; alleles of 8/8, 9/58 and 12/138 repeat) and patient (*n* = 3; alleles of 291/315, 9/597 and 16/402 repeat) iPSCs remained unchanged across serial passages (Supplementary Fig. 7).

### Analysis of the *FGF14* GAA•TTC repeat somatic instability in post-mortem brain tissues

Because transcription through GAA•TTC repeat tracts enhances somatic instability,^27^ the very low expression levels of *FGF14* in blood and fibroblasts^1^ may account for the minimal instability of the repeat in these tissues. In comparison, *FGF14* is widely expressed in the CNS, with the highest expression levels observed in the cerebellum.^7^ Specifically, *FGF14* isoform 1b (*FGF14*-201, transcript 2; ENST00000376131.9; NM_175929.3), within which the GAA•TTC repeat is located in its first intron, has notably higher expression levels in the cerebellum compared to isoform 1a (*FGF14*-202, transcript 1; ENST00000376143.5; NM_004115.4) according to the Genotype-Tissue Expression (GTEx) Project database. We therefore hypothesized that higher degrees of somatic instability may be present in the CNS. To address this question, we conducted a comprehensive analysis of the somatic instability of *FGF14* GAA•TTC repeat tracts in multiple post-mortem CNS regions from six non-ataxic controls and six patients with SCA27B (Table 1).

**Figure 3:**
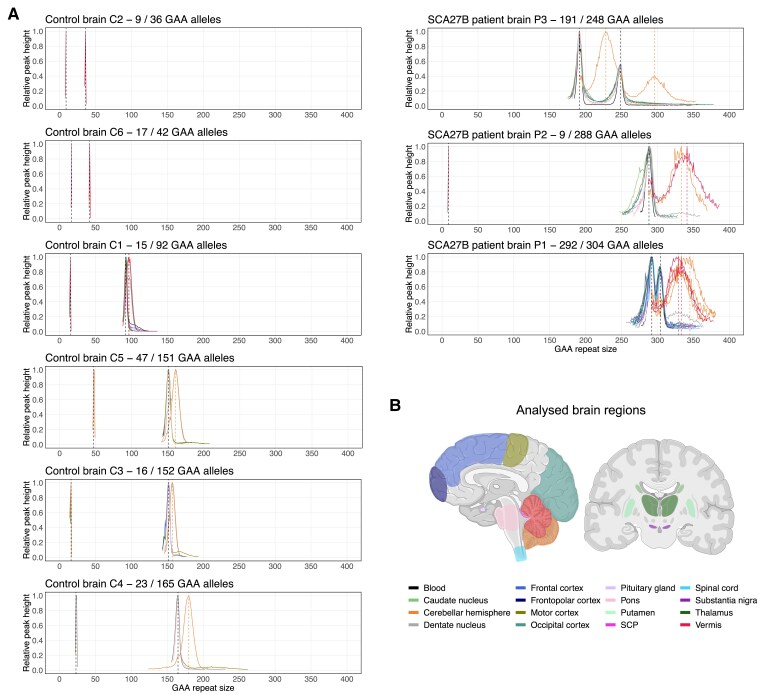
**Somatic instability profile of the *FGF14* GAA•TTC repeat in brain regions**. (**A**) Somatic instability profiles of the *FGF14* GAA•TTC repeat in different brain regions, derived from post-mortem samples of six non-ataxic control and three patients with SCA27B. Each plot shows the average instability profile for each of the two alleles within a given brain region, calculated from replicate PCR reactions. For regions where multiple tissue samples were analysed, results for each sample are shown individually. Profiles were plotted by normalizing individual peak height data to the height of the modal allele within each brain region, except for SCA27B cases P1 and P3, where data were normalized to the height of the shorter modal allele due to the lack of significant size difference with the longer allele. Peaks left of the modal allele above a 10% threshold and those right of the modal allele above a 1% threshold were plotted. Vertical dashed black lines indicate the size of the modal alleles measured in the blood and/or non-cerebellar regions, while vertical dashed red and orange lines indicate the size of the modal alleles measured in the vermis and cerebellar hemispheres, respectively. Individual instability profiles in each region are shown in Supplementary Figs 8–16. (**B**) Schematic representation of the various brain regions analysed for somatic instability in this study. The same colour scheme is used to represent the brain regions and their corresponding instability profiles shown in **A**. Panel created with BioRender.com.

**Figure 4:**
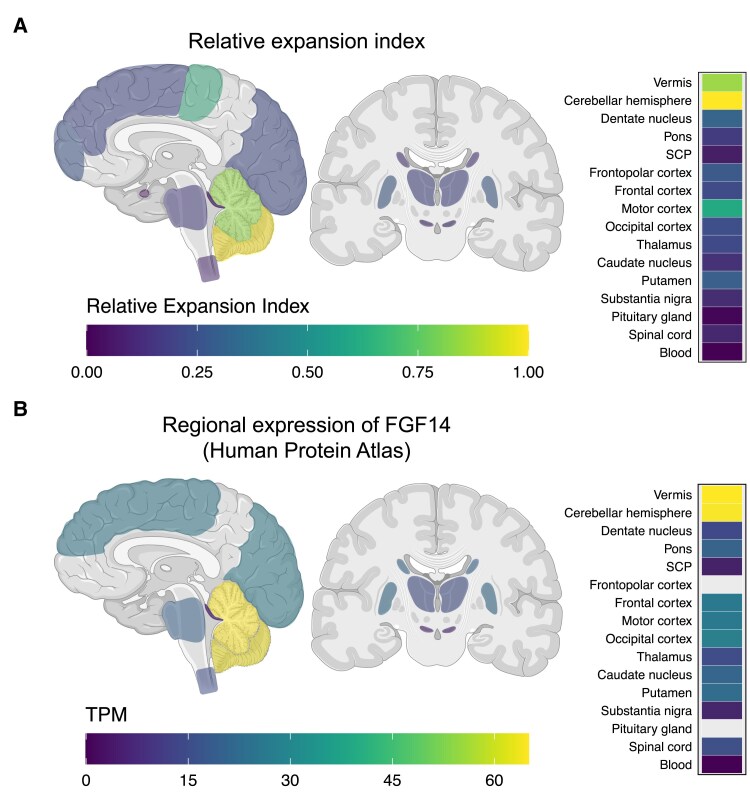
**Expansion index of the *FGF14* GAA•TTC repeat in brain regions**. Schematic representation of (**A**) the relative expansion index and (**B**) the regional expression of *FGF14* normal tissue expression data in transcripts per million (TPM) from the Human Protein Atlas across the analysed brain regions shown in Fig. 3. Expansion indices for each brain region were normalized relative to those in the cerebellar hemispheres. The relative expansion indices were calculated by averaging the relative expansion index of each allele, excluding (GAA)_9_ alleles, which did not exhibit somatic expansion. A corresponding heat map of the results for each region is shown on the *right*. Expression data were not available for the frontopolar cortex and the pituitary gland. Figure created with BioRender.com.

In controls C1–C6 and patients P1P3, all of whom carried alleles smaller than 400 GAA•TTC triplets, analysis of tissue-wide somatic instability in multiple brain regions revealed that the modal allele length and EI of both wild-type and expanded alleles were remarkably stable across all analysed regions, except in the cerebellar hemispheres and vermis, which showed a marked somatic expansion bias (Figs 3 and 4A; see also Supplementary material, ‘Results’ section). In control and patient brains, the modal length of wild type and expanded alleles was identical—within two triplets—across all non-cerebellar regions and blood samples, while, for alleles longer than ≈90 repeats, it increased on average by 10% ± 6% in the vermis and 11% ± 6% in the cerebellar hemispheres (Fig. 3A and Supplementary Figs 17–25). Levels of somatic expansions, as measured by the EI, were markedly increased in the cerebellar hemispheres and vermis compared to other CNS regions (Fig. 4A). Excluding (GAA)_9_ alleles, which did not exhibit somatic expansion, the EI in the cerebellar hemispheres was, on average, 3.23 times greater relative to other CNS regions outside the cerebellum. Furthermore, within the cerebellum itself, the EI in the hemisphere was 1.15 times greater relative to the vermis and 2.42 times greater relative to the dentate nucleus. We found that increasing allele length was associated with higher degrees of instability of the *FGF14* repeat locus, with instability even observed in alleles as short as 15 triplets, indicating that somatic instability is not exclusive to large, pathogenic *FGF14* alleles (Supplementary Fig. 26). The pattern of somatic expansion in the CNS was closely correlated with the regional expression of *FGF14*, which is highest in the cerebellar hemispheres and vermis (Fig. 4). This observation supports the hypothesis that higher levels of transcription through GAA•TTC repeat tracts promote their somatic expansion.^27^ Furthermore, we found a positive correlation between the EI and the repeat length in many of the examined tissues, with the steepest regression slopes observed in the vermis [r = 0.96 (95% CI: 0.81–0.99), slope = 0.130, *P* < 0.0001] and the cerebellar hemispheres [r = 0.93 (95% CI: 0.81–0.98), slope = 0.133, *P* < 0.0001] (Supplementary Fig. 26). Using a multiple regression model, we found significant relationships between EI and repeat size (estimate = 0.047, SE = 0.005, *P* < 0.0001), tissue expression (estimate = 0.133, SE = 0.026, *P* < 0.0001) and female sex (estimate = 6.630, SE = 1.744, *P* = 0.00022), although the latter association should be interpreted cautiously as it was based on only two female cases. In contrast, age at death did not have a significant effect on EI (estimate = 0.040, SE = 0.061, *P* = 0.51). These variables together explained approximately 58% of the variance in EI. Overall, our results demonstrate that the *FGF14* repeat locus exhibits a strong somatic expansion bias largely restricted to the cerebellum, that may in part be driven by the high level of *FGF14* expression in this tissue. This observation contrasts with a recent study on SCA1, SCA2, SCA3 and SCA7, which showed that despite high *ATXN1*, *ATXN2*, *ATXN3* and *ATXN7* gene expression, the cerebellum exhibited the lowest degree of somatic mosaicism.^14^

**Figure 5:**
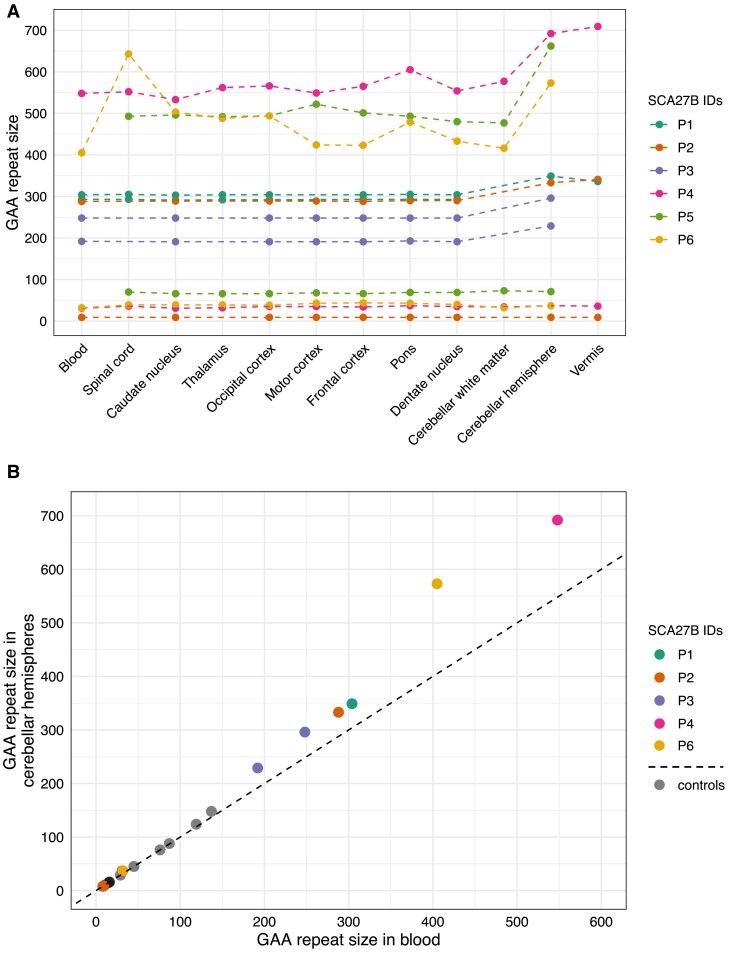
***FGF14* GAA•TTC repeat lengths in brain regions of SCA27B patients**. (**A**) Distribution of *FGF14* GAA•TTC repeat lengths, expressed in triplet repeat counts, across post-mortem brain regions and blood samples in the six SCA27B patients included in this study. For brain regions where multiple tissue samples were analysed, only the results of the sample with the largest allele size are shown. Observations for each of the two alleles from the same patient are connected by a dashed line. (**B**) Comparison of *FGF14* GAA•TTC repeat lengths in blood samples (*x*-axis) versus cerebellar hemispheres (*y*-axis) for five SCA27B patients and eight controls with available tissue samples. The dashed black line represents the identity line. In **A** and **B**, a single modal allele size is shown for patient P1 due to the complete blending of the instability profiles for the short and long alleles in the cerebellar hemispheres and vermis (Supplementary Fig. 33), preventing the identification of the two distinct modal peaks in these tissues.

We also studied the somatic instability of the *FGF14* repeats by agarose gel electrophoresis in the post-mortem brains of three patients with SCA27B (P4-P6), each carrying expansions longer than 400 triplets. This analysis confirmed the cerebellar-specific somatic expansion bias of the *FGF14* repeat locus. Compared to other tissues, the expansion length in the cerebellar hemispheres was 87–159 triplets longer in patient P4, 129–185 triplets longer in patient P5 and 70–168 triplets longer in patient P6 (Fig. 5A and Supplementary Figs 53–55). Furthermore, compared to blood samples, this corresponds to an increase in expansion length in the cerebellar hemispheres of 26% in patient P4 and 41% in patient P6 (Fig. 5B). Among the three patients, cerebellar vermis was only available for study in patient P4 and exhibited a greater expansion length compared to the cerebellar hemispheres (709 versus 692 GAA•TTC repeats). Of note, in keeping with the greater levels of *FGF14* expression in cerebellar neurons compared to glia,^26^ the cerebellar hemispheres and vermis showed longer expansion sizes relative to the cerebellar white matter in all three patients. Finally, although the cervical spinal cord exhibited the largest expansion size of all tissues examined from patient P6, this finding was not replicated in the two other patients (P1 and P5) for whom cervical spinal cord was available for study. This discrepancy could be due to differences in the specific regions of the spinal cord sampled for analysis.

Comparison of modal lengths in blood samples and cerebellar hemispheres from five patients with SCA27B (P1, P2, P3, P4, P6) and eight control individuals (C7–C14) revealed that sizes were largely stable in both tissues for alleles shorter than ≈90 repeat units. However, for longer alleles, the modal length in the cerebellar hemispheres increased with allele size [r = 0.99 (95% CI: 0.96–1.00), slope = 1.365, *P* < 0.0001] (Fig. 5B). Remarkably, this threshold of ≈90 repeat units for instability in the cerebellar hemispheres is highly similar to the threshold of 75–100 repeat units for instability during intergenerational transmission.^4,8^ Despite the similar instability thresholds, different mechanisms are likely responsible for instability in the cerebellum and the germline, given the comparatively lower *FGF14* expression in testes and ovaries and the repeat contraction observed with paternal transmission.^4,8^

### Methylation profiling of *FGF14* in post-mortem cerebellum

**Figure 6:**
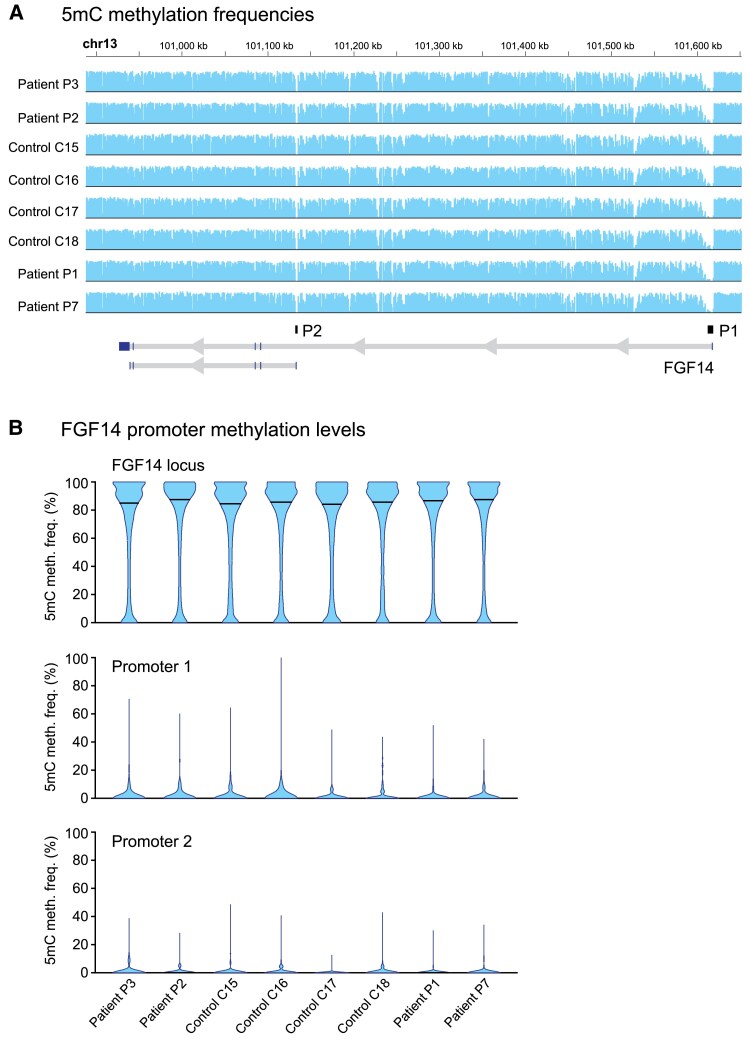
**Methylation profiling of the *FGF14* gene in post-mortem cerebellum**. Methylation analysis of the *FGF14* gene in post-mortem cerebellar hemispheres from four controls (C15–C18) and four patients with SCA27B (P1, P2, P3, P7) using programmable targeted long-read sequencing with Oxford Nanopore Technologies. (**A**) Methylation profiles, expressed as 5′-methylcytosine (5mC) methylation frequencies, at all CpG sites within the *FGF14* locus (T2T-CHM13, chr13:100923763-101619864) in the four control and four patient post-mortem cerebellar hemispheres. A diagram of the *FGF14* gene with its two putative promoters (P1: promoter 1 and P2: promoter 2) is shown at the *bottom*. (**B**) Plots show the distribution of 5mC methylation frequencies at all CpG sites within the *FGF14* locus (*top*), the first promoter (*middle*) and second promoter (*bottom*). The horizontal black bars show the median 5mC methylation frequency.

Preliminary investigations of patient-derived post-mortem cerebellum and iPSC-derived neurons have suggested that the intronic GAA•TTC repeat expansion likely leads to loss of function of isoform 1b (transcript 2) by interfering with *FGF14* transcription.^1^ Since the *FGF14* expansion shares the same repeat motif as the Friedreich’s ataxia *FXN* expansion (GAA•TTC), which is known to cause hypermethylation of the DNA upstream of the expanded repeat locus (differentially methylated region),^28^ we investigated whether altered methylation was present in post-mortem cerebellum from four patients with SCA27B. Using programmable targeted ONT long-read sequencing,^29^ we found no evidence of significant difference in 5mC methylation frequencies of *FGF14* [median frequency (IQR) in patients versus controls, 69.75 (68.65–69.80) versus 68.95 (67.43–69.80); Mann–Whitney U-test, *P* = 0.63] and its two putative promoters [first promoter: 2.00 (1.35–2.27) versus 2.25 (1.40–3.25); Mann–Whitney U-test, *P* = 0.54; and second promoter: 0.85 (0.57–1.35) versus 0.80 (0.40–0.90); Mann–Whitney U-test, *P* = 0.63] in controls compared to patients (Fig. 6). The region surrounding the repeat expansion (10 kb window centred on the repeat locus) showed slightly higher CpG methylation compared to the region surrounding wild-type alleles, although the difference was not statistically significant [median frequency (IQR) in expanded alleles versus wild-type alleles, 82.37 (79.96–84.76) versus 78.78 (76.66–80.55); Mann–Whitney U-test, *P* = 0.11]. These results do not exclude the possibility that the expansion is associated with hypermethylation of *FGF14*, which will require further study in a larger number of cases.

## Discussion

This study comprehensively assessed the somatic instability of the *FGF14* GAA•TTC repeat in peripheral tissues and post-mortem brains of patients with SCA27B and uncovered a marked tendency for the repeat to somatically expand in the cerebellar vermis and hemispheres. Our findings provide new insight into the biology of SCA27B, potentially explaining the selective vulnerability of the cerebellum—specifically the grey matter—and the relative resilience of other regions in this disease.^1,3^ Although somatic expansion events were detected in all tissues analysed, the expansion bias was considerably higher in the cerebellar hemispheres and vermis, which are the primary sites of neuronal loss in SCA27B.

Although brain mosaicism has only been studied in a few spinocerebellar ataxias, previous studies of SCA1, SCA2, SCA3, SCA7 and DRPLA have consistently shown that their causative coding CAG•CTG repeat expansions exhibit smaller sizes and lower degrees of mosaicism in the cerebellum compared to other brain regions.^13-17^ Studies of Huntington’s disease, myotonic dystrophy type 1 and spinal and bulbar muscular atrophy, all caused by CAG•CTG repeat expansions, have also shown that the smallest expansions and lowest somatic instability occur in the cerebellum.^13,30-32^ In comparison, a recent study found that the pathogenic *DAB1* intronic ATTTC repeat insertion causing SCA37 exhibits longer sizes and greater somatic expansion in the cerebellum compared to blood samples and fibroblasts of two patients,^18^ suggesting that the instability pattern of CAG•CTG repeat expansions in the cerebellum may differ from that observed with other types of repeats. The factors underlying the distinct behaviour of the *FGF14* GAA•TTC repeat expansion in the cerebellum compared to CAG•CTG repeat expansions remain unknown, although they may involve *cis*-acting elements, differential effects of mismatch repair pathways on CAG•CTG repeats compared to GAA•TTC repeats, or an increased ability of the long and uninterrupted *FGF14* GAA•TTC repeat to form alternative DNA structures that can become substrates for DNA repair machineries.^33^

In Friedreich’s ataxia, although the expanded *FXN* GAA•TTC repeat is longer in the cerebellum than in other CNS regions, large contractions occur significantly more frequently than large expansions in the cerebellum.^20-23^ Progressive expansion of the *FXN* repeat over time has been described in human cells, cerebellum, and other terminally differentiated tissues in both human and mouse models.^20-23,34,35^ DNA repair has been established as a major factor modulating GAA•TTC repeat stability.^9,33^ Studies in a non-dividing quiescent *Saccharomyces cerevisiae* model have shown that large contractions of the GAA•TTC repeat expansion may be driven by non-homologous end joining repair of DNA breaks of the repeat mediated by mismatch repair complexes.^36^ Furthermore, activity of the mismatch repair complex MutSβ has been shown to promote *FXN* GAA•TTC expansion in Friedreich’s ataxia patient-derived cells.^37,38^ Similarly, DNA repair mechanisms may also be involved in somatic expansion of the *FGF14* GAA•TTC repeat, especially considering the high expression levels of mismatch repair genes in the cerebellum compared to other brain regions.^14^ Moreover, 5′ GAA-strand and 5′ TTC-strand nicks (single-strand breaks) have recently been shown to induce expansion of GAA•TTC repeats in non-dividing yeast cells,^39^ raising the hypothesis that such phenomenon may contribute to somatic instability in post-mitotic neurons, which are known to accumulate single-strand breaks over time.^40^

Our findings suggest that tissue-specific transcriptional activity through the GAA•TTC repeat promotes its instability, which align with a previous study on GAA•TTC repeat instability in human cell lines.^27^ The GAA•TTC repeat is located within intron 1 of *FGF14* isoform 1b, the isoform predominantly expressed in the CNS, particularly in the cerebellum.^7^ Analysis of tissue-wide somatic instability revealed that the pattern of somatic expansion in the CNS closely correlates with the regional expression of *FGF14*. The cerebellar vermis and hemispheres, which have the highest and second highest levels of *FGF14* expression in the CNS, respectively, exhibited the greatest somatic expansion across all tissues analysed. Moreover, the association between *FGF14* repeat instability and expression levels is further corroborated by our results showing longer expansions in the cerebellar hemispheres and vermis compared to the cerebellar white matter, in keeping with the substantially greater expression of *FGF14* in cerebellar neurons relative to glia.^26^ Alternative DNA structures called DNA triplexes are formed in expanded GAA•TTC repeats in Friedreich’s ataxia patient-derived cells,^41^ and transcription through these repeats has been shown to induce the formation of triplexes, which may fuel repeat instability.^9,42^ This observation contrasts with a recent study of the somatic instability of CAG•CTG repeats, which are prone to form DNA hairpins,^43^ but not DNA triplexes, in the polyglutamine spinocerebellar ataxias SCA1, SCA2, SCA3 and SCA7. These pathogenic CAG•CTG repeats showed lower levels of expansion in the cerebellum despite high expression levels of the *ATXN1*, *ATXN2*, *ATXN3* and *ATXN7* genes.^14^ The absence of significant somatic instability of the CAG•CTG repeat in the cerebellum, which is one of the most pathologically affected brain regions in these spinocerebellar ataxias,^44^ further supports the possibility that different alternative DNA structures and various repair pathways may differentially impact the stability of CAG•CTG repeats compared to GAA•TTC repeats in the cerebellum.

Although we observed no change in repeat size and EI in blood samples over time, somatic expansions of the *FGF14* mutant alleles in the cerebellar cortical neurons are likely to progress with age, with the rate of expansion increasing with the number of repeats. Indeed, we found that increasing allele length was associated with higher degrees of instability of the *FGF14* GAA•TTC repeat locus. While our study did not look at mosaicism in foetal brains, somatic instability is likely to progress throughout life rather than arise during CNS development (as previously shown in Friedreich’s ataxia^20^ and Huntington’s disease,^45^ for example), as regions with the same embryological origin, such as the cerebellar cortex, dentate nuclei and pons, all deriving from the metencephalon,^46^ present dramatically different instability patterns. This hypothesis would also be consistent with the late age at onset of SCA27B, suggesting that the disease may only manifest when a critical proportion of cerebellar Purkinje cells accumulate expansions above a cell-specific pathogenic threshold, which is likely to be higher than the currently used ‘blood-based threshold’.^47^ It also raises the possibility that loss of function of *FGF14* isoform 1b may only occur when repeat sizes extend beyond this specific threshold in cerebellar Purkinje cells, which have the highest *FGF14* expression.^48^ However, since Purkinje cells constitute only about 0.1% of cerebellar neurons, they contribute minimally to the instability readout in bulk cerebellar tissue. The possibility of Purkinje cells having a higher cell-specific pathogenic threshold would be further supported by the observation that despite the majority of granule cells—the most abundant cerebellar cortical neurons and primary contributors to the instability readout—exhibiting somatic instability, they are only mildly affected in patients with SCA27B.^1,3^ This hypothesis also provides a potential explanation for the incomplete penetrance of certain *FGF14* expansions, suggesting that asymptomatic carriers may accumulate somatic expansions in Purkinje cells at lower rates compared to symptomatic individuals, preventing them from accumulating a sufficiently large burden of somatic expansions to manifest the disease. Furthermore, variable somatic expansion, which may be influenced by variations in DNA repair genes, may underlie the significant phenotypic variability among patients carrying expansions of similar sizes.

Preliminary functional studies in post-mortem cerebellum and iPSC-derived motor neurons from patients with SCA27B suggest that the intronic GAA•TTC repeat expansion leads to transcriptional deficiency and partial loss of function of the *FGF14* isoform 1b.^1^ Although the mechanisms through which the expanded GAA•TTC repeat in *FGF14* causes disease remain to be established, it may involve similar epigenetic mechanisms as in Friedreich’s ataxia, which is the only other known disease caused by intronic GAA•TTC repeat expansions.^49^ The expanded GAA•TTC repeat in Friedreich’s ataxia results in transcriptional deficiency of the frataxin (*FXN*) gene.^50^ Transcriptional deficiency in Friedreich’s ataxia is caused by various mechanisms, including the formation of DNA triplexes and RNA/DNA hybrids (R-loops) by the expanded GAA•TTC repeat, hypermethylation of the DNA upstream of the expanded repeat, and heterochromatin-mediated repression resulting from hypoacetylation of histones H3K9, H3K4, and H4K5 and trimethylation of histone H3K9 and H3K27.^28,41,51-59^ Although we found that the region surrounding the expanded GAA•TTC repeat in *FGF14* exhibited slightly higher levels of methylation compared to the wild-type allele, this difference did not reach statistical significance. The absence of statistically significant difference may be attributed to the analysis of bulk tissue rather than isolated Purkinje cells, which are the primary cells affected in SCA27B.^1,3^ Additionally, whether other epigenetic mechanisms involved in Friedreich’s ataxia, such as heterochromatin-mediated transcriptional repression, also occur in SCA27B will deserve further investigation.

Our study has several strengths, including the inclusion of a large number of post-mortem controls and SCA27B cases with a variety of allele sizes, the availability of serial blood samples for many patients over long periods of time, and the ability to accurately size and calculate expansion indices in samples carrying expansions shorter than ≈400–450 triplets. However, our results need to be interpreted in light of some limitations. First, cerebellar vermis, which is the most pathologically affected region in SCA27B,^1,3^ was available for analysis in only half of the patients and controls, thus limiting the analysis of somatic instability in this region. Second, the inability of capillary electrophoresis to resolve low-abundance alleles and alleles longer than ≈400–450 triplets prevented a comprehensive analysis of the full spectrum of allele length variability in the cerebellum. Analysis of bulk PCR products by capillary electrophoresis will preferentially detect shorter GAA•TTC tracts and cannot capture the full extent of GAA•TTC repeat expansions in any given tissue. Third, our analyses did not permit the detection of rare, very large expansion events that may be occurring in Purkinje cells, which are primarily affected in SCA27B. Purkinje cells make up only a small fraction of the total cerebellar cell population and, as such, do not contribute significantly to the instability readout measured in bulk tissue. Additional techniques, such as single-cell sequencing, will be necessary to dissect cell-specific somatic instability in SCA27B. Fourth, in post-mortem tissues from patients with neurodegenerative ataxia, it is unknown whether the genotype of degenerated cerebellar neurons is similar to that of the remaining cells. However, it is possible that neurons carrying longer expansions degenerate quickly, leading to an underrepresentation of longer allele sizes in the instability readout.

In conclusion, our analysis of the *FGF14* GAA•TTC repeat somatic instability in post-mortem brain samples from individuals with SCA27B revealed a marked expansion bias in the cerebellar hemispheres and vermis. Our study offers new insights into the biology of SCA27B by providing a potential explanation for the significant phenotypic variability and the incomplete penetrance of certain expansions. It also opens up important new lines of inquiry for understanding the mechanisms driving somatic instability of tandem repeats, the cell-specific patterns of instability and their relationship to neurodegeneration.

## Supplementary Material

awae312_Supplementary_Data

## Data Availability

The data supporting this study may be shared at the request of any qualified investigator on reasonable request.

## References

[1] Pellerin D, Danzi MC, Wilke C, et al Deep intronic FGF14 GAA repeat expansion in late-onset cerebellar ataxia. N Engl J Med. 2023;388:128–141. 36516086 10.1056/NEJMoa2207406 PMC10042577

[2] Rafehi H, Read J, Szmulewicz DJ, et al An intronic GAA repeat expansion in FGF14 causes the autosomal-dominant adult-onset ataxia SCA27B/ATX-FGF14. Am J Hum Genet. 2023;110:1018. 37267898 10.1016/j.ajhg.2023.05.005 PMC10257192

[3] Wilke C, Pellerin D, Mengel D, et al GAA-FGF14 ataxia (SCA27B): Phenotypic profile, natural history progression and 4-aminopyridine treatment response. Brain. 2023;146:4144–4157. 37165652 10.1093/brain/awad157

[4] Méreaux JL, Davoine CS, Pellerin D, et al Clinical and genetic keys to cerebellar ataxia due to FGF14 GAA expansions. EBioMedicine. 2024;99:104931. 38150853 10.1016/j.ebiom.2023.104931 PMC10784672

[5] Pellerin D, Heindl F, Wilke C, et al GAA-FGF14 disease: Defining its frequency, molecular basis, and 4-aminopyridine response in a large downbeat nystagmus cohort. EBioMedicine. 2024;102:105076. 38507876 10.1016/j.ebiom.2024.105076 PMC10960126

[6] Pellerin D, Danzi MC, Renaud M, et al Spinocerebellar ataxia 27B: A novel, frequent and potentially treatable ataxia. Clin Transl Med. 2024;14:e1504. 38279833 10.1002/ctm2.1504 PMC10819088

[7] Wang Q, McEwen DG, Ornitz DM. Subcellular and developmental expression of alternatively spliced forms of fibroblast growth factor 14. Mech Dev. 2000;90:283–287. 10640713 10.1016/s0925-4773(99)00241-5

[8] Pellerin D, Del Gobbo GF, Couse M, et al A common flanking variant is associated with enhanced stability of the FGF14-SCA27B repeat locus. Nat Genet. 2024;56:1366–1370. 38937606 10.1038/s41588-024-01808-5 PMC11440897

[9] Khristich AN, Mirkin SM. On the wrong DNA track: Molecular mechanisms of repeat-mediated genome instability. J Biol Chem. 2020;295:4134–4170. 32060097 10.1074/jbc.REV119.007678 PMC7105313

[10] Jones L, Houlden H, Tabrizi SJ. DNA repair in the trinucleotide repeat disorders. Lancet Neurol. 2017;16:88–96. 27979358 10.1016/S1474-4422(16)30350-7

[11] Matos-Rodrigues G, Hisey JA, Nussenzweig A, Mirkin SM. Detection of alternative DNA structures and its implications for human disease. Mol Cell. 2023;83:3622–3641. 37863029 10.1016/j.molcel.2023.08.018 PMC12673489

[12] Depienne C, Mandel JL. 30 years of repeat expansion disorders: What have we learned and what are the remaining challenges? Am J Hum Genet. 2021;108:764–785. 33811808 10.1016/j.ajhg.2021.03.011 PMC8205997

[13] Mouro Pinto R, Arning L, Giordano JV, et al Patterns of CAG repeat instability in the central nervous system and periphery in Huntington's disease and in spinocerebellar ataxia type 1. Hum Mol Genet. 2020;29:2551–2567. 32761094 10.1093/hmg/ddaa139 PMC7471505

[14] Kacher R, Lejeune FX, David I, et al CAG repeat mosaicism is gene specific in spinocerebellar ataxias. Am J Hum Genet. 2024;111:913–926. 38626762 10.1016/j.ajhg.2024.03.015 PMC11080609

[15] Matsuura T, Sasaki H, Yabe I, et al Mosaicism of unstable CAG repeats in the brain of spinocerebellar ataxia type 2. J Neurol. 1999;246:835–839. 10525984 10.1007/s004150050464

[16] Cancel G, Gourfinkel-An I, Stevanin G, et al Somatic mosaicism of the CAG repeat expansion in spinocerebellar ataxia type 3/Machado-Joseph disease. Hum Mutat. 1998;11:23–27. 9450899 10.1002/(SICI)1098-1004(1998)11:1<23::AID-HUMU4>3.0.CO;2-M

[17] Takano H, Onodera O, Takahashi H, et al Somatic mosaicism of expanded CAG repeats in brains of patients with dentatorubral-pallidoluysian atrophy: Cellular population-dependent dynamics of mitotic instability. Am J Hum Genet. 1996;58:1212–1222. 8651298 PMC1915058

[18] Sanchez-Flores M, Corral-Juan M, Gasch-Navalón E, Cirillo D, Sanchez I, Matilla-Dueñas A. Novel genotype-phenotype correlations, differential cerebellar allele-specific methylation, and a common origin of the (ATTTC)_n_ insertion in spinocerebellar ataxia type 37. Hum Genet. 2024;143:211–232. 38396267 10.1007/s00439-024-02644-7 PMC11043136

[19] Currò R, Dominik N, Facchini S, et al Role of the repeat expansion size in predicting age of onset and severity in RFC1 disease. Brain. 2024;147:1887–1898. 38193360 10.1093/brain/awad436 PMC11068103

[20] De Biase I, Rasmussen A, Monticelli A, et al Somatic instability of the expanded GAA triplet-repeat sequence in Friedreich ataxia progresses throughout life. Genomics. 2007;90:1–5. 17498922 10.1016/j.ygeno.2007.04.001

[21] De Biase I, Rasmussen A, Endres D, et al Progressive GAA expansions in dorsal root ganglia of Friedreich's ataxia patients. Ann Neurol. 2007;61:55–60. 17262846 10.1002/ana.21052

[22] Long A, Napierala JS, Polak U, et al Somatic instability of the expanded GAA repeats in Friedreich's ataxia. PLoS One. 2017;12:e0189990. 29261783 10.1371/journal.pone.0189990 PMC5736210

[23] Montermini L, Kish SJ, Jiralerspong S, Lamarche JB, Pandolfo M. Somatic mosaicism for Friedreich's ataxia GAA triplet repeat expansions in the central nervous system. Neurology. 1997;49:606–610. 9270608 10.1212/wnl.49.2.606

[24] Lee JM, Zhang J, Su AI, et al A novel approach to investigate tissue-specific trinucleotide repeat instability. BMC Syst Biol. 2010;4:29. 20302627 10.1186/1752-0509-4-29 PMC2856555

[25] Bonnet C, Pellerin D, Roth V, et al Optimized testing strategy for the diagnosis of GAA-FGF14 ataxia/spinocerebellar ataxia 27B. Sci Rep. 2023;13:9737. 37322040 10.1038/s41598-023-36654-8 PMC10272173

[26] Sjöstedt E, Zhong W, Fagerberg L, et al An atlas of the protein-coding genes in the human, pig, and mouse brain. Science. 2020;367:eaay5947. 32139519 10.1126/science.aay5947

[27] Ditch S, Sammarco MC, Banerjee A, Grabczyk E. Progressive GAA.TTC repeat expansion in human cell lines. PLoS Genet. 2009;5:e1000704. 19876374 10.1371/journal.pgen.1000704 PMC2760145

[28] Rodden LN, Chutake YK, Gilliam K, et al Methylated and unmethylated epialleles support variegated epigenetic silencing in Friedreich ataxia. Hum Mol Genet. 2021;29:3818–3829. 33432325 10.1093/hmg/ddaa267 PMC7861014

[29] Stevanovski I, Chintalaphani SR, Gamaarachchi H, et al Comprehensive genetic diagnosis of tandem repeat expansion disorders with programmable targeted nanopore sequencing. Sci Adv. 2022;8:eabm5386. 35245110 10.1126/sciadv.abm5386 PMC8896783

[30] Kennedy L, Evans E, Chen CM, et al Dramatic tissue-specific mutation length increases are an early molecular event in Huntington disease pathogenesis. Hum Mol Genet. 2003;12:3359–3367. 14570710 10.1093/hmg/ddg352

[31] Jinnai K, Mitani M, Futamura N, Kawamoto K, Funakawa I, Itoh K. Somatic instability of CTG repeats in the cerebellum of myotonic dystrophy type 1. Muscle Nerve. 2013;48:105–108. 23629807 10.1002/mus.23717

[32] Tanaka F, Reeves MF, Ito Y, et al Tissue-specific somatic mosaicism in spinal and bulbar muscular atrophy is dependent on CAG-repeat length and androgen receptor–gene expression level. Am J Hum Genet. 1999;65:966–973. 10486315 10.1086/302578 PMC1288267

[33] Masnovo C, Lobo AF, Mirkin SM. Replication dependent and independent mechanisms of GAA repeat instability. DNA Repair (Amst). 2022;118:103385. 35952488 10.1016/j.dnarep.2022.103385 PMC9675320

[34] Clark RM, De Biase I, Malykhina AP, Al-Mahdawi S, Pook M, Bidichandani SI. The GAA triplet-repeat is unstable in the context of the human FXN locus and displays age-dependent expansions in cerebellum and DRG in a transgenic mouse model. Hum Genet. 2007;120:633–640. 17024371 10.1007/s00439-006-0249-3

[35] Al-Mahdawi S, Pinto RM, Ruddle P, Carroll C, Webster Z, Pook M. GAA repeat instability in Friedreich ataxia YAC transgenic mice. Genomics. 2004;84:301–310. 15233994 10.1016/j.ygeno.2004.04.003

[36] Neil AJ, Hisey JA, Quasem I, et al Replication-independent instability of Friedreich's ataxia GAA repeats during chronological aging. Proc Natl Acad Sci U S A. 2021;118:e2013080118. 33495349 10.1073/pnas.2013080118 PMC7865128

[37] Du J, Campau E, Soragni E, et al Role of mismatch repair enzymes in GAA·TTC triplet-repeat expansion in Friedreich ataxia induced pluripotent stem cells. J Biol Chem. 2012;287:29861–29872. 22798143 10.1074/jbc.M112.391961 PMC3436184

[38] Halabi A, Ditch S, Wang J, Grabczyk E. DNA mismatch repair complex MutSβ promotes GAA·TTC repeat expansion in human cells. J Biol Chem. 2012;287:29958–29967. 22787155 10.1074/jbc.M112.356758 PMC3436174

[39] Li L, Scott WS, Khristich AN, Armenia JF, Mirkin SM. Recurrent DNA nicks drive massive expansions of (GAA)_n_ repeats. Proc Natl Acad Sci U S A. 2024; 121:e2413298121. 39585990 10.1073/pnas.2413298121 PMC11626148

[40] Wu W, Hill SE, Nathan WJ, et al Neuronal enhancers are hotspots for DNA single-strand break repair. Nature. 2021;593:440–444. 33767446 10.1038/s41586-021-03468-5 PMC9827709

[41] Matos-Rodrigues G, van Wietmarschen N, Wu W, et al S1-END-seq reveals DNA secondary structures in human cells. Mol Cell. 2022;82:3538–3552.e5. 36075220 10.1016/j.molcel.2022.08.007 PMC9547894

[42] Neil AJ, Liang MU, Khristich AN, Shah KA, Mirkin SM. RNA-DNA hybrids promote the expansion of Friedreich's ataxia (GAA)_n_ repeats via break-induced replication. Nucleic Acids Res. 2018;46:3487–3497. 29447396 10.1093/nar/gky099 PMC5909440

[43] Pearson CE, Tam M, Wang YH, et al Slipped-strand DNAs formed by long (CAG)*(CTG) repeats: Slipped-out repeats and slip-out junctions. Nucleic Acids Res. 2002;30:4534–4547. 12384601 10.1093/nar/gkf572 PMC137136

[44] Rüb U, Schöls L, Paulson H, et al Clinical features, neurogenetics and neuropathology of the polyglutamine spinocerebellar ataxias type 1, 2, 3, 6 and 7. Prog Neurobiol. 2013;104:38–66. 23438480 10.1016/j.pneurobio.2013.01.001

[45] Kacher R, Lejeune FX, Noël S, et al Propensity for somatic expansion increases over the course of life in Huntington disease. Elife. 2021;10:e64674. 33983118 10.7554/eLife.64674 PMC8118653

[46] Later Development of Embryonic Central Nervous System . Reference module in biomedical sciences. Elsevier; 2014.

[47] Kaplan S, Itzkovitz S, Shapiro E. A universal mechanism ties genotype to phenotype in trinucleotide diseases. PLoS Comput Biol. 2007;3:e235. 18039028 10.1371/journal.pcbi.0030235 PMC2082501

[48] Shakkottai VG, Xiao M, Xu L, et al FGF14 regulates the intrinsic excitability of cerebellar Purkinje neurons. Neurobiol Dis. 2009;33:81–88. 18930825 10.1016/j.nbd.2008.09.019 PMC2652849

[49] Campuzano V, Montermini L, Moltò MD, et al Friedreich's ataxia: Autosomal recessive disease caused by an intronic GAA triplet repeat expansion. Science. 1996;271:1423–1427. 8596916 10.1126/science.271.5254.1423

[50] Delatycki MB, Bidichandani SI. Friedreich ataxia- pathogenesis and implications for therapies. Neurobiol Dis. 2019;132:104606. 31494282 10.1016/j.nbd.2019.104606

[51] Sakamoto N, Chastain PD, Parniewski P, et al Sticky DNA: Self-association properties of long GAA.TTC repeats in R.R.Y triplex structures from Friedreich's ataxia. Mol Cell. 1999;3:465–475. 10230399 10.1016/s1097-2765(00)80474-8

[52] Ohshima K, Montermini L, Wells RD, Pandolfo M. Inhibitory effects of expanded GAA.TTC triplet repeats from intron I of the Friedreich ataxia gene on transcription and replication in vivo. J Biol Chem. 1998;273:14588–14595. 9603975 10.1074/jbc.273.23.14588

[53] Greene E, Mahishi L, Entezam A, Kumari D, Usdin K. Repeat-induced epigenetic changes in intron 1 of the frataxin gene and its consequences in Friedreich ataxia. Nucleic Acids Res. 2007;35:3383–3390. 17478498 10.1093/nar/gkm271 PMC1904289

[54] Saveliev A, Everett C, Sharpe T, Webster Z, Festenstein R. DNA triplet repeats mediate heterochromatin-protein-1-sensitive variegated gene silencing. Nature. 2003;422:909–913. 12712207 10.1038/nature01596

[55] Soragni E, Herman D, Dent SY, Gottesfeld JM, Wells RD, Napierala M. Long intronic GAA*TTC repeats induce epigenetic changes and reporter gene silencing in a molecular model of Friedreich ataxia. Nucleic Acids Res. 2008;36:6056–6065. 18820300 10.1093/nar/gkn604 PMC2577344

[56] Li Y, Lu Y, Polak U, et al Expanded GAA repeats impede transcription elongation through the FXN gene and induce transcriptional silencing that is restricted to the FXN locus. Hum Mol Genet. 2015;24:6932–6943. 26401053 10.1093/hmg/ddv397 PMC4654050

[57] Groh M, Lufino MM, Wade-Martins R, Gromak N. R-loops associated with triplet repeat expansions promote gene silencing in Friedreich ataxia and fragile X syndrome. PLoS Genet. 2014;10:e1004318. 24787137 10.1371/journal.pgen.1004318 PMC4006715

[58] Chutake YK, Costello WN, Lam C, Bidichandani SI. Altered nucleosome positioning at the transcription start site and deficient transcriptional initiation in Friedreich ataxia. J Biol Chem. 2014;289:15194–15202. 24737321 10.1074/jbc.M114.566414 PMC4140879

[59] Yandim C, Natisvili T, Festenstein R. Gene regulation and epigenetics in Friedreich's ataxia. J Neurochem. 2013;126(Suppl 1):21–42. 23859339 10.1111/jnc.12254

